# Enhancing Enzyme-Mediated Cellulose Hydrolysis by Incorporating Acid Groups Onto the Lignin During Biomass Pretreatment

**DOI:** 10.3389/fbioe.2020.608835

**Published:** 2020-11-13

**Authors:** Jie Wu, Richard P. Chandra, Masatsugu Takada, Li-Yang Liu, Scott Renneckar, Kwang Ho Kim, Chang Soo Kim, Jack N. Saddler

**Affiliations:** ^1^Forest Product Biotechnology/Bioenergy Group, Department of Wood Science, Faculty of Forestry, University of British Columbia, Vancouver, BC, Canada; ^2^International Advanced Energy Science Research and Education Center, Graduate School of Energy Science, Kyoto University, Kyoto, Japan; ^3^Advanced Renewable Materials Lab, Department of Wood Science, Faculty of Forestry, University of British Columbia, Vancouver, BC, Canada; ^4^Clean Energy Research Center, Korea Institute of Science and Technology, Seoul, South Korea

**Keywords:** lignin, oxidation, sulfonation, cellulase enzymes, non-productive binding, pH

## Abstract

Lignin is known to limit the enzyme-mediated hydrolysis of biomass by both restricting substrate swelling and binding to the enzymes. Pretreated mechanical pulp (MP) made from Aspen wood chips was incubated with either 16% sodium sulfite or 32% sodium percarbonate to incorporate similar amounts of sulfonic and carboxylic acid groups onto the lignin (60 mmol/kg substrate) present in the pulp without resulting in significant delignification. When Simon’s stain was used to assess potential enzyme accessibility to the cellulose, it was apparent that both post-treatments enhanced accessibility and cellulose hydrolysis. To further elucidate how acid group addition might influence potential enzyme binding to lignin, Protease Treated Lignin (PTL) was isolated from the original and modified mechanical pulps and added to a cellulose rich, delignified Kraft pulp. As anticipated, the PTLs from both the oxidized and sulfonated substrates proved less inhibitory and adsorbed less enzymes than did the PTL derived from the original pulp. Subsequent analyses indicated that both the sulfonated and oxidized lignin samples contained less phenolic hydroxyl groups, resulting in enhanced hydrophilicity and a more negative charge which decreased the non-productive binding of the cellulase enzymes to the lignin.

## Introduction

Lignin has been proven to be a significant obstacle when trying to carry out effective enzyme-mediated hydrolysis of lignocellulosic substrates. Past work has shown that this lignin inhibits hydrolysis by the two major mechanisms its presence limiting substrate swelling, consequently restricting cellulose accessibility, as well lignin binding to the cellulolytic enzymes and limiting their activities (Nakagame et al., 2010; Del Rio et al., 2011; Kumar et al., 2012; Rahikainen et al., 2013). Although lignin removal has been shown to be an effective way to enhance enzyme-mediated cellulose hydrolysis (Mooney et al., 1998), to date, no cost-effective delignification-based methods have been commercialized, primarily due to the cost associated with the use of chemicals (Takada et al., 2020). As a result, a considerable amount of work has focused on trying to mitigate lignins inhibitory effect by modifying it, such as using relatively mild reaction conditions that incorporate acid groups onto the lignin macromolecule, consequently enhancing cellulose hydrolysis without the need for complete delignification (Eriksson et al., 2002; Berlin et al., 2006; Pan, 2008; Del Rio et al., 2011; Nakagame et al., 2011a).

Oxidation and sulfonation have been the most common methods used as this modifies the lignin structure and charge by the incorporation of acid groups. Both approaches are widely used by the pulp and paper sector, with alkaline oxidation predominantly used to remove residual lignin from unbleached Kraft pulp (Fengel and Wegener, 1989; Kalliola et al., 2011) and sulfonation predominantly used to soften the lignin prior to mechanically pulping wood chips (Börås and Gatenholm, 1999). More recently, oxidation and sulfonation treatments, which result in the incorporation of carboxylic and sulfonic acid groups, respectively, have been incorporated into pretreatment methods to enhance accessibility to the cellulose while retaining the hemicellulose and lignin components (Chandra et al., 2016; Chu et al., 2017; Wu et al., 2019, 2020). For example, incorporating acid groups onto the lignin has been shown to both enhance fiber swelling, consequently increasing enzyme accessibility to the cellulose (Del Rio et al., 2011), as well as reducing lignin-enzyme interaction resulting from hydrophobic (Eriksson et al., 2002), ionic (Berlin et al., 2006), and hydrogen bond interactions (Sewalt et al., 1997; Pan, 2008). As a result of these treatments the lignin also becomes more hydrophilic, reducing the tendency for it to hydrophobically bind to enzymes (Nakagame et al., 2011a). The deprotonation of the acid groups generates a negative charge, facilitating the electrostatic repulsion between the negatively charged lignin and cellulase enzymes (Nakagame et al., 2010; Del Rio et al., 2011).

Despite these insights the benefits of acid group integration on biomass deconstruction have yet to be fully resolved. For example, how the structure and characteristic of the sulfonic (strong acid) and carboxylic (weak acid) acids influences cellulose hydrolysis (Fengel and Wegener, 1989; Ben et al., 1993; Yang et al., 2003) while, at the same time, raising the pH level might have the potential to further ionize the acid groups attached to the lignin. As this has been shown to increase the hydrophilicity and negative charge, it might also facilitate the repulsion between lignin and the negatively charged cellulases (Lan et al., 2013; Lou et al., 2013).

In the work reported here, similar amounts of carboxylic and sulfonic acid groups were incorporated onto the lignin present in pretreated mechanical pulps. The research described in the manuscript helped better elucidate the beneficial effect of sulfonic and carboxylic acid group addition to lignin in terms of enzyme accessibility/deconstruction to/off cellulose. Both the oxidized and sulfonated lignin samples were shown to be less inhibitory to cellulose hydrolysis and also adsorbed less enzymes. The sulfonated and oxidized lignin contained more acid groups and aliphatic hydroxyl groups and less phenolic hydroxyl groups. This enhanced lignin hydrophilicity and increased the negative charge, decreasing the non-productive binding of cellulases to lignin.

## Materials and Methods

### Biomass and Chemicals

Aspen wood chips were obtained from a pulp mill in Western Canada and the chips were screened using a 2.5 × 2.5 cm and 5.0 × 5.0 cm mesh. During the water-based pretreatment, the chips were heated at 170°C for 1 h, according to Wu et al. (2019). The pretreated Aspen was subsequently subject to mechanical pulping/refining using a twin-gear juicer (super angel juicer model 8500) in a total volume of 10 L of water. The acid wash of the pretreated Aspen mechanical pulp (MP) was conducted according to Meng et al. (2015). The cellulose-rich delignified Kraft pulp was donated by Fortress Ltd. The protease enzymes, sodium percarbonate, sodium sulfite and anhydrous ether were all purchased from Millipore Sigma (Oakville, Canada).

### Alkaline Oxidation and Neutral Sulfonation of Pretreated Aspen

Twenty gram of pretreated and acid-washed mechanical pulp was incubated overnight with either 16% sodium sulfite or 32% sodium percarbonate at a10% solid loading in a 60°C water bath. The substrates were washed with water after the reactions and stored at 4°C for further analyses.

### Enzymatic Hydrolysis

Cellic CTec 3 cellulase mixture used for enzymatic hydrolysis has a protein content of 208.9 mg/mL and cellulase activity of FPU/mL. The enzymatic hydrolysis of the substrates at a 2% solids loading was performed in 2 mL screwcap centrifuge tubes containing sodium acetate buffer (50 mM, pH 4.8 and pH 6) and the Cellic CTec 3 (in duplicate). The tubes were incubated in a rotating incubator at 50°C and the released sugars measured using a glucose analyzer (YSI 2700 Select Biochemistry Analyzer) after 6, 24, and 48 h of hydrolysis. Prior to enzymatic hydrolysis, the protein content of the Cellic CTec 3 preparation was measured using the ninhydrin essay, according to Mok et al. (2015).

### Substrate Characterizations

The chemical composition of the substrates was analyzed according to the Klason protocol using TAPPI standard method T222, as described by Wu et al. (2019). The accessibility of the cellulose to enzymes was measured by the modified Simon’s stain method using the high molecular weight fraction of Direct Orange 15 dye, according to Chandra and Saddler (2012). The conductometric titration of acid groups was conducted using a modified version of the method developed by Katz and Beatson (1984).

### Protease Treated Lignin (PTL) Isolation

The PTL was isolated from the biomass substrates using the method previously described by Nakagame (2010). In brief, the pretreated substrates were subjected to enzymatic hydrolysis using the Cellic CTec 3 preparation at 2% solids and protein loading of 30 mg/g cellulose at 50°C for 72 h. The hydrolysis residue was centrifuged, washed and subjected to a second round of enzymatic hydrolysis using the same conditions, followed by centrifugation and washing. This residue was incubated with protease (1 U/mL) in phosphate buffer (50 mM, pH 7) at 37°C for 24 h and subsequently transferred to a 90°C water bath for 2 h to deactivate the protease. After washing, the protease treated lignin (PTL) was passed through a 40-mesh screen and freeze-dried.

### Enzymatic Mild Acidolysis Lignin (EMAL) Isolation

The EMAL lignin was extracted from the protease treated lignin (PTL) samples. In brief, the freeze-dried PTL was subject to mild acidolysis in an acidic aqueous dioxane solution (85% Dioxane: 15% water, 0.01M HCl) under reflux at 87°C for 3 h. The mixture was then centrifuged (5,000 rpm, 5 min) and the supernatant collected and precipitated in anhydrous ether. After the lignin had settled it was filtered through a polyvinylidene fluoride (PVDF) membrane, washed with anhydrous ether and dried in a 40°C vacuum oven.

### Hydrophobicity Test of Lignin

The hydrophobicity of the protease treated lignin (PTL) samples was estimated by their adoption to Rose Bengal dye, according to Huang et al. (2017).

### Zeta Potential Measurement of Lignin

The Zeta potential values were determined in triplicate using a Zeta-Meter 3.0 + (ZETA-METER, INC., Staunton, VA). Samples were dispersed in 50 mM Na-acetate buffer (pH 4.8 and pH 6) before measurement.

### Adsorption of Lignin to Advanced Enzyme Cocktail (Cellic CTec3)

The adsorption of the enzymes present in the Cellic CTec3 to the isolated protease treated lignin (PTL) samples was performed at 10°C in 2.0 mL crew-cap centrifuge tubes using 1ml of sodium acetate buffer (50 mM, pH 4.8 and pH 6). Vials containing 1% (w/v) lignin and enzymes (0.5 mg/mL) were incubated for 3 h, followed by centrifugation and the supernatant collected. The protein content of the supernatants was measured by the ninhydrin method according to Mok et al. (2015), using BSA as the protein standard. The amount of enzyme adsorbed onto the lignin was determined as the difference between the initial enzyme loading and the free enzyme present in the supernatant.

### Gel Permeation Chromatography (GPC)

The molecular weight of EMAL lignin samples was analyzed by GPC using an Agilent 1100 equipped with an optilab T-rEX differential refractive index detector (dRI, Wyatt Tech. CA, United States) and poly(styrenesulfonate) as standard and DMSO/LiBr (0.5% w/v) as eluent at a flow rate of 0.5 ml/min. Prior to analysis, 10 mg of lignin was dissolved in 1 ml DMSO/BrLi (0.5% w/v), left at room temperature for 48 h and filtered through 0.45 μm PTFE filters. The data were collected and analyzed using ASTRA 6.0 software.

### ^31^P Nuclear Magnetic Resonance (^31^P NMR)

The types and amount of hydroxyl groups located on the EMAL lignin was analyzed by ^31^P NMR using a Bruker Avance 300 MHz spectrometer, according to the method described by Song et al. (2019). Chromium (III) acetylacetonate and N-hydroxy-5- norbornene-2,3-dicarboximide were selected as the respective relaxation reagent and internal standard and they were dissolved in pyridine/CDCl_3_ mixture (1.6:1, v/v) at concentrations of 5.6 and 9.0 mg/mL, respectively. 20 mg of dried EMAL lignin was dissolved in 400 μL of pyridine/CDCl_3_ mixture, where 100 μL of internal standard solution, 40 μL of relaxation reagent solution, and 50 μL of 2-chloro- 4,4,5,5-tetramethyl-1,2,3-dioxaphospholane (TMDP) were added prior to ^31^P NMR analysis. An inverse gated decoupling pulse was used with the following parameters; number of scans 800, relaxation delay 5 s, acquisition time 1.4 s, pulse length 6 μs, and 90° pulse width.

## Results and Discussion

### The Influence of Lignin Sulfonation and Oxidation on Cellulose Accessibility and Enzymatic Hydrolysis

As we anticipated that the presence of hemicellulose and the inherent acid groups (e.g., cinnamic acids and acetyl groups) associated with the biomass might also influence substrate swelling (Sjöström et al., 1965; Ju et al., 2013) and cellulose hydrolysis, a pretreatment step was first carried out at 170°C, to primarily solubilize the hemicellulose while minimizing lignin condensation (Garrote et al., 2001; Li et al., 2010). The pretreated Aspen slurry was subsequently pulped using a twin-gear juicer to simulate mechanical pulping. As initial conductometric titration had indicated that the pretreated mechanical pulp (MP) still contained some weak acids, the pulp was subsequently acid washed to remove any residual uronic acid groups that were potentially located on the residual hemicellulose (Meng et al., 2015). After this series of treatments, the pulps contained less than 10% hemicellulose and any associated acid groups were below detectable levels (Table 1). The subsequent alkaline oxidation and neutral sulfonation reactions of acid group-free pulp was carried out using sodium percarbonate (Na_2_CO_3_ ⋅ 1.5 H_2_O_2_) and sodium sulfite (Na_2_SO_3_), respectively, as alkaline oxidation and neutral sulfonation had previously been shown to enhance the enzymatic hydrolysis of pretreated pulps (Yang et al., 2002; Kumar et al., 2011). As we hoped to modify the lignin rather than remove it, the reaction was carried out at 60°C to minimize solubilizing the more hydrophilic oxidized/sulfonated lignin. As summarized in Table 1, a chemical loading of 16% sodium sulfite and 32% sodium percarbonate resulted in the incorporation of similar amounts of sulfonic and carboxylic acid groups (60 mmol/kg) while recovering more than 90% of the original water-insoluble component (Table 1). As described earlier Sjöström et al. (1965), sulfonation also increased the exposure of carboxylic acid groups, likely due to the cleavage of ester linkages between lignin and hemicellulose.

**TABLE 1:** Chemical composition and characteristics of unmodified, sulfonated and oxidized mechanical pulps (MP) derived from pretreated Aspen chips.

**Sample**	**Pretreatment yield (%)**	**Chemical composition**			**Substrate characteristics**			
		**Glucan (%)**	**Xylan (%)**	**Lignin (%)**	**Sulfonic acid groups (mmol/kg)**	**Carboxylic acid groups (mmol/kg)**	**Total acid groups (mmol/kg)**	**DO dye adsorption (mg/g dry substrate)**
Unmodified MP	100.0	63 ± 1	7 ± 0	30 ± 0	0 ± 0	0 ± 0	0 ± 0	84
Sulfonated MP	93.0	67 ± 2	4 ± 1	29 ± 0	44 ± 6	20 ± 5	64 ± 3	93
Oxidized MP	91.6	67 ± 0	5 ± 0	29 ± 0	0 ± 0	59 ± 1	59 ± 1	95

**FIGURE 1:**
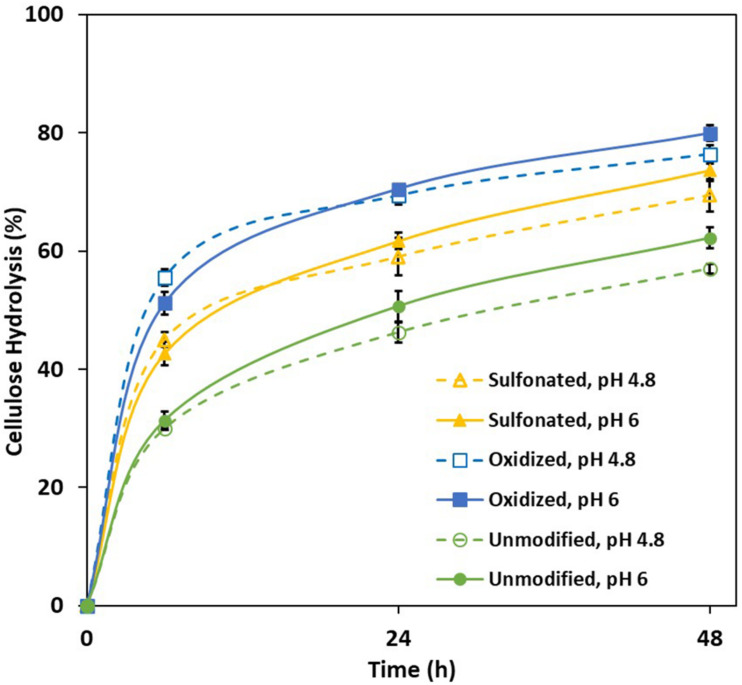
Extent of enzyme-mediated hydrolysis of the cellulose present in unmodified, sulfonated and oxidized mechanical pulps (MP) at 2% solids and enzyme loading of 20 mg/g cellulose.

Earlier work had shown that acid group addition to the lignin enhanced substrate swelling, resulting in both increased enzyme accessibility and hydrolysis of the cellulose (Del Rio et al., 2011). As the Simon’s stain method has been successfully used (Stone et al., 1969; Yu and Atalla, 1998) to predict the cellulose accessibility to cellulase enzymes (Chandra et al., 2009) by measuring substrate’s adsorption to Directed Orange (DO) dye, when this method was used it was apparent that sulfonation and oxidation both increased the accessibility of all three pretreated mechanical pulps (Table 1), likely due to enhanced substrate swelling resulting from lignin modification. It was also apparent that the sulfonation and oxidation treatments enhanced enzyme-mediated cellulose hydrolysis (Figure 1) with the oxidized substrate being more susceptible, despite the fact that both pulps had similar amounts of lignin and acid groups (Table 1). However, as has been suggested previously (Sjöström et al., 1965), it is likely that the majority of the carboxylic acid groups present in the sulfonated substrate were located on the hemicellulose component, while the carboxylic acid groups associated with the oxidized substrate were associated more with the lignin.

**FIGURE 2:**
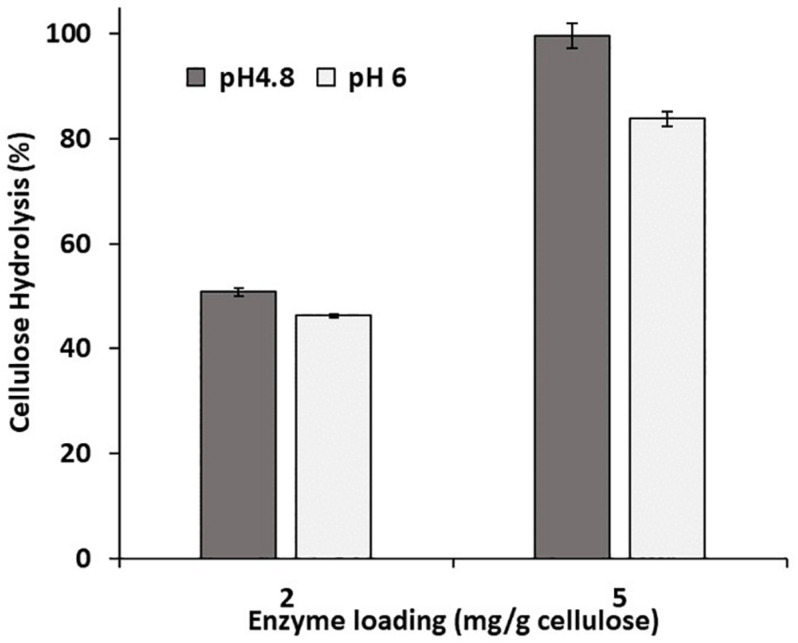
Enzymatic hydrolysis of a cellulose-rich, delignified Kraft pulp at 2% solids, pH 4.8 and 6, and an enzyme loading of 2 and 5 mg/g cellulose. Enzymatic hydrolysis was performed for 48 h in a 50°C rotating incubator.

Previous work had suggested that hydrolysis could be enhanced at an elevated pH by further deprotonating the acid groups and consequently increasing the lignin’s hydrophilicity and negative charge (Lan et al., 2013; Lou et al., 2013). However, the hydrolysis of both the control and modified substrates was only slightly enhanced when it was carried out at pH 6 (Figure 1). To ensure that activity of the cellulases was not compromised by the use of an elevated pH, a cellulose-rich delignified Kraft pulp was hydrolyzed at both pH 4.8 and pH 6 (Figure 2). As observed previously, it was apparent that slightly better hydrolysis was achieved at pH 4.8, (Reese and Mandels, 1980; Ayyachamy et al., 2013).

### The Isolation, Characterization and Influence of Control and Modified Lignin on Enzymatic Hydrolysis

**FIGURE 3:**
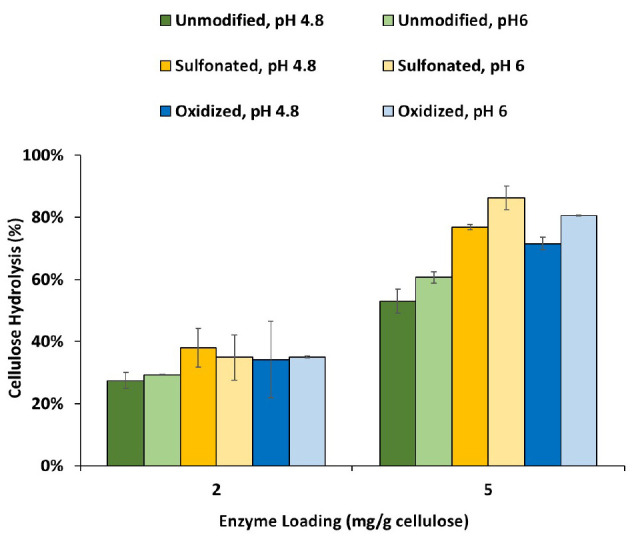
Enzymatic hydrolysis of cellulose-rich delignified Kraft pulp with added PTLs isolated from unmodified, sulfonated and oxidized mechanical pulps (MP) at 2% solids and enzyme loading of 2 and 5 mg/g cellulose. Enzymatic hydrolysis was performed for 48h in a 50°C rotating incubator.

As previous work had shown that lignin inhibits enzyme-mediated hydrolysis of cellulose by both limiting the swelling of the substrate and binding with the enzymes, we next tried to differentiate how sulfonation and oxidation might influence these two mechanisms. As it is difficult to extract lignin from biomass substrates without modifying it, Protease Treated Lignin (PTL) has often been used to represent the lignin present in pretreated biomass (Nakagame et al., 2011b; Yuan et al., 2018). This method uses a cellulase cocktail to remove the carbohydrate components to produce a lignin-rich residue which is subsequently treated with protease to remove the enzymes. Despite the removal of much of the carbohydrate by the enzyme treatment, it should be noted that some residual material was still associated with the lignin (Table 2). When the modified PTL’s were add to the hydrolysis of a cellulose-rich Kraft pulp (at 1:1 ratio), at both pH 4.8 and 6 (Figure 3), it was apparent that the modified PTLs were both less inhibitory with better cellulose hydrolysis achieved at pH 6. When the adsorption isotherms of the cellulases enzyme in the presence of the PTLs were measured at pH 4.8 and pH 6, it was apparent that sulfonation and oxidation both decreased the extent of adsorption between the enzymes and the modified lignin (Table 2). Although somewhat unexpected, it is likely that the observed increase in hydrolysis observed at the higher pH was due to the decreased non-productive binding of the enzymes to the lignin.

**TABLE 2:** Chemical composition, potential adsorption of cellulases, hydrophobicity (assessed by Rose Bengal adsorption) and negative charge (assessed by Zeta Potential) of lignins isolated from unmodified, sulfonated, and oxidized mechanical pulps (MP).

**Sample**	**Chemical composition**		**Binding strength at pH 4.8 (mL/g lignin)**	**Binding strength at pH 6 (mL/g lignin)**	**Hydrophobicity (L/g)**	**Zeta potential at pH 4.8**	**Zeta potential at pH 6**
	**Glucan (%)**	**Lignin (%)**					
Unmodified MP	30 ± 2	70 ± 1	95.2	87.0	1.14	6 ± 0	0 ± 1
Sulfonated MP	28 ± 1	74 ± 2	80.6	54.9	0.80	−6 ± 1	−8 ± 1
Oxidized MP	29 ± 1	72 ± 2	74.1	47.6	0.46	0 ± 1	−6 ± 3

As acid groups addition is known to influence both the hydrophilicity and negative charge of the lignin, consequently decreasing enzyme binding (Nakagame et al., 2011b), we next wanted to confirm these earlier observations using Rose Bengal method and Zeta potential measurements (Nakagame et al., 2011b; Huang et al., 2017; Song et al., 2019). It was apparent that sulfonic and carboxylic acid group addition to the lignin significantly enhanced its hydrophilicity and negative charge (Table 2) with the decrease in the Zeta potential of the oxidized PTL at pH 6 indicating more carboxylic acid group disassociation.

**FIGURE 4:**
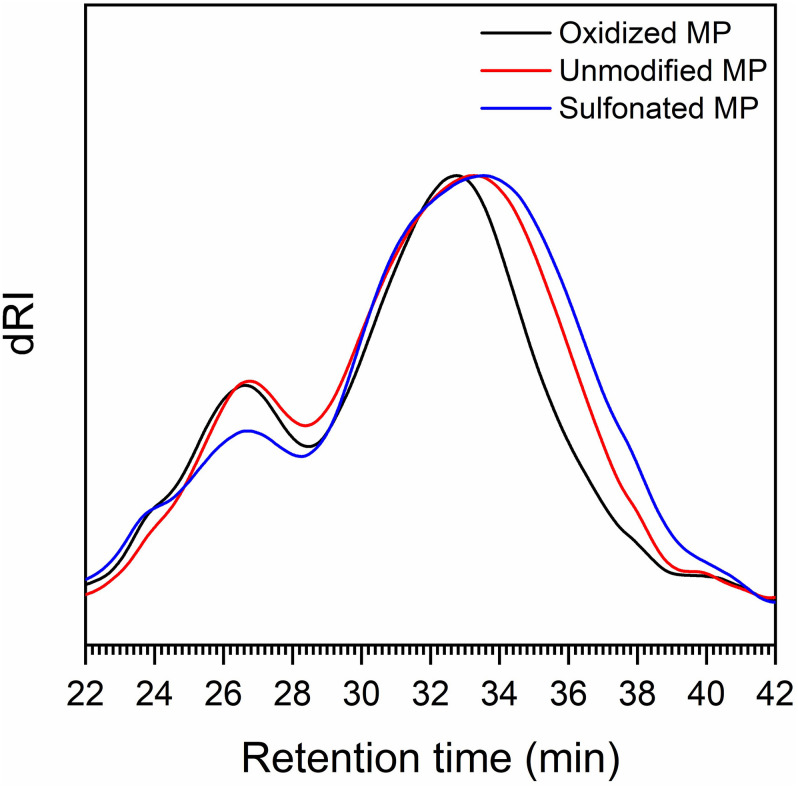
GPC profile of the lignin extracted from unmodified, sulfonated and oxidized pre-hydrolyzed mechanical pulps (MP).

As it was possible that the residual cellulose associated with each of the PTL samples might influence lignin analysis and its structure, we also used the Enzymatic Mild Acidolysis Lignin (EMAL) method to isolate lignin at relatively high yields without changing its structure and properties (Guerra et al., 2006). Subsequent Gel Permeation Chromatography (GPC) analysis of the EMAL samples indicated that both sulfonation and oxidation resulted in a slight increase in the molecular weight of the lignin components, suggesting that the lignin underwent mild re-polymerization reaction under alkali conditions (Das et al., 2019; Table 3 and Figure 4). Previous work has used ^31^P Nuclear Magnetic Resonance (NMR) analysis to successfully quantify the amount and type of hydroxyl groups (–OH) located on the aromatic and aliphatic structures of lignin (Pu et al., 2011). As previous studies had indicated that Syringyl lignin is much easier to remove than Guaiacyl lignin, during the pretreatment of hardwood biomass (Yu et al., 2014; Li et al., 2016), it was likely that a small amount of syringyl subunit lignin was removed during oxidation and sulfonation (Table 1). Interestingly, both sulfonation and oxidation resulted in a slight increase in the aliphatic hydroxyl group content of the lignin which was probably due to the cleavage of the ester groups connecting the lignin aliphatic chain and carbohydrates during the sulfonation and oxidation of lignin (Crestini and Argyropoulos, 1997). The observed increase in the carboxylic acid groups attached to the EMAL lignin further confirmed that lignin oxidation had occurred (Table 3). When the observed changes in the hydroxyl group content of the lignin was compared to the extent of cellulose hydrolysis, it was apparent that those substrates whose lignin contained more aliphatic hydroxyl groups and less phenolic hydroxyl groups were more easily hydrolyzed (Figures 1, 3 and Table 3). This was consistent with previous studies which had suggested that the aliphatic hydroxyl groups in the lignin made it more hydrophilic, thereby reducing enzyme binding. In contrast, a greater amount of phenolic hydroxyl groups appeared to enhance enzyme binding (Li et al., 2016; Yang and Pan, 2016).

**TABLE 3:** Molecular weight and hydroxyl group content of the lignin extracted from unmodified, sulfonated and oxidized pre-hydrolyzed mechanical pulps (MP).

**Sample**	**Molecular Weight**			**Hydroxyl groups as determined by ^31^P NMR (mmol/g)**					
		
	**Mn (kDa)**	**Mw (kDa)**	**PDI**	**Aliphatic −OH**	**Syringyl phenolic −OH**	**Guaiacyl phenolic −OH**	**p-Hydroxy phenyl phenolic −OH**	**Total phenolic −OH**	**−COOH**
Unmodified MP	2.3	47.5	20.9	4.19	1.11	0.61	0.40	2.12	0.10
Sulfonated MP	3.1	60.6	19.4	4.35	0.94	0.50	0.39	1.82	0.05
Oxidized MP	1.4	53.5	38.0	4.53	0.84	0.59	0.37	1.80	0.19

## Conclusion

Lignin is known to inhibit effective enzyme-mediated hydrolysis of biomass by both restricting substrate swelling and by binding with cellulase enzymes. Both enzyme accessibility and cellulose hydrolysis could be enhanced by the addition of sulfonic and carboxylic acid groups onto the lignin present in biomass substrates. It was apparent that both oxidation and sulfonation decreased the extent of enzyme binding with the reduction in the number of phenolic hydroxyl groups as well as the incorporation of acid groups enhancing lignin hydrophilicity, consequently enhancing cellulose hydrolysis.

## Data Availability Statement

The original contributions presented in the study are included in the article/supplementary material, further inquiries can be directed to the corresponding authors.

## Author Contributions

JW, RC, MT, and JS designed the study. JW wrote the manuscript with the support from RC and JS. JW performed the experiments. L-YL, SR, KK, and CK contributed to the experimental section. All authors contributed to the article and approved the submitted version.

## Conflict of Interest

The authors declare that the research was conducted in the absence of any commercial or financial relationships that could be construed as a potential conflict of interest.
