# Corrigendum: Enhancing enzyme-mediated cellulose hydrolysis by incorporating acid groups onto the lignin during biomass pretreatment

**DOI:** 10.3389/fbioe.2024.1545045

**Published:** 2025-01-07

**Authors:** Jie Wu, Richard P. Chandra, Masatsugu Takada, Li-Yang Liu, Scott Renneckar, Kwang Ho Kim, Chang Soo Kim, Jack N. Saddler

**Affiliations:** ^1^ Forest Product Biotechnology/Bioenergy Group, Department of Wood Science, Faculty of Forestry, University of British Columbia, Vancouver, BC, Canada; ^2^ International Advanced Energy Science Research and Education Center, Graduate School of Energy Science, Kyoto University, Kyoto, Japan; ^3^ Advanced Renewable Materials Lab, Department of Wood Science, Faculty of Forestry, University of British Columbia, Vancouver, BC, Canada; ^4^ Clean Energy Research Center, Korea Institute of Science and Technology, Seoul, Republic of Korea

**Keywords:** lignin, oxidation, sulfonation, cellulase enzymes, non-productive binding, pH

In the published article, there was an error in Figure 3 as published. During the initial submission process, an incorrect image of Figure 3 (showing the hydrolysis results using a less advanced cellulase mixture) was accidentally used during the final stage. This cellulase mixture lacks accessory enzymes, resulting in lower hydrolysis yields at elevated pH. In contrast, the more advanced enzyme cocktail CTec3, used in this study, contains accessory enzymes that reduce non-productive lignin-enzyme binding at elevated pH, as described in the main content of the article. The corrected Figure 3 and its caption appear below.

**FIGURE 3:**
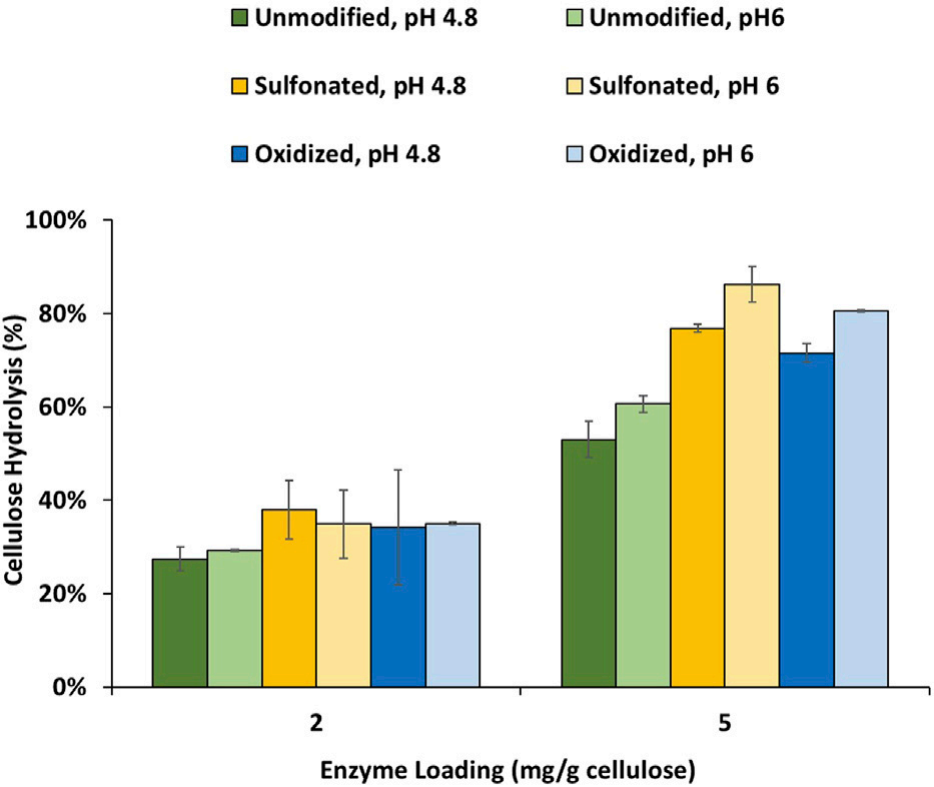
Enzymatic hydrolysis of cellulose-rich delignified Kraft pulp with added PTLs isolated from unmodified, sulfonated and oxidized mechanical pulps (MP) at 2% solids and enzyme loading of 2 and 5 mg/g cellulose. Enzymatic hydrolysis was performed for 48 h in a 50°C rotating incubator.

The authors apologize for this error and state that this does not change the scientific conclusions of the article in any way. The original article has been updated.

